# Supplementary material for: Whole-Genome Sequencing of 502 Individuals from Latvia: The First Step towards a Population-Specific Reference of Genetic Variation
Source: Int J Mol Sci. 2023 Oct 19;24(20):15345. doi: 10.3390/ijms242015345 (PMC10607061; doi:10.3390/ijms242015345)
Supplement: Supplementary file 1 [file Supplementary Figure S1.pdf › Supplementary Figure S1.pdf]

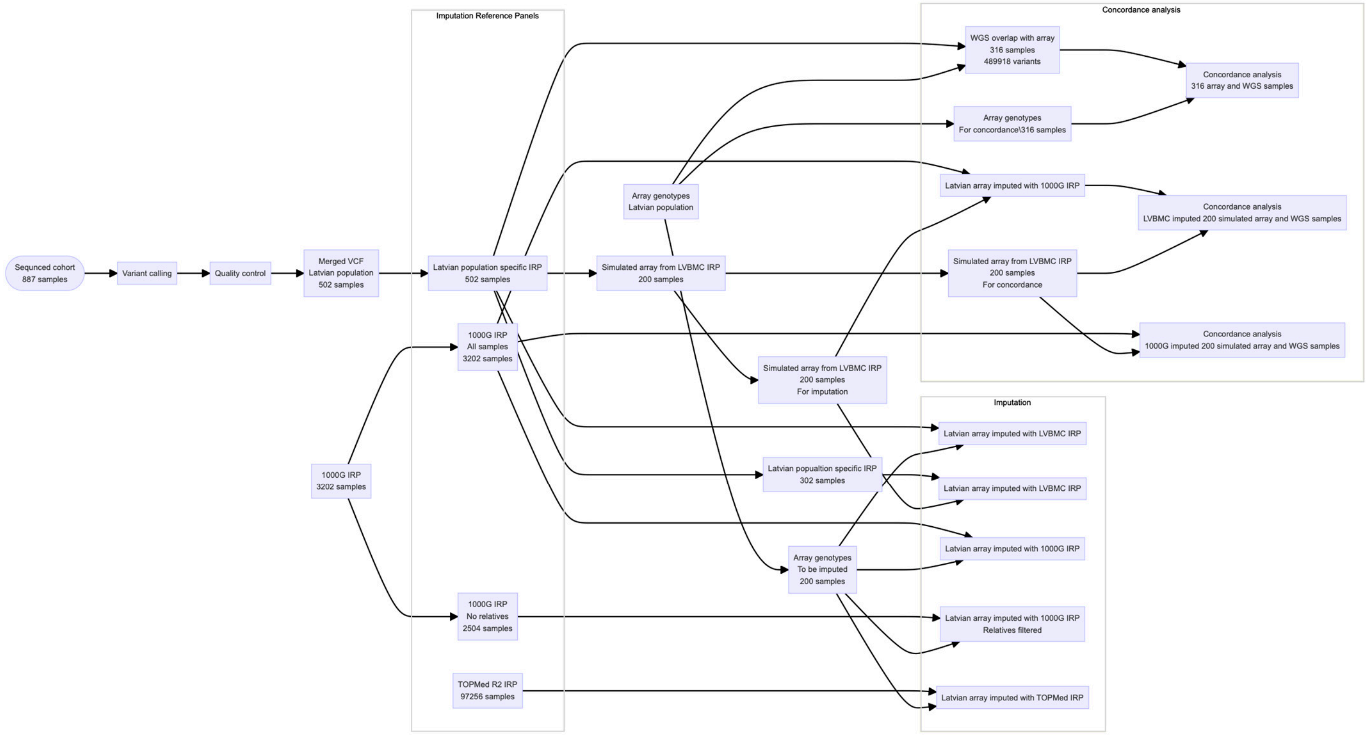

Figure S1: Flowchart of initial data preparation, imputation, and concordance analysis.
